# TDP-43 promotes the formation of neuromuscular synapses through the regulation of Disc-large expression in Drosophila skeletal muscles

**DOI:** 10.1186/s12915-020-00767-7

**Published:** 2020-03-26

**Authors:** Nina Strah, Giulia Romano, Clelia Introna, Raffaella Klima, Marta Marzullo, Laura Ciapponi, Aram Megighian, Monica Nizzardo, Fabian Feiguin

**Affiliations:** 1 International Centre for Genetic Engineering and Biotechnology, Padriciano 99, 34149 Trieste, Italy; 2 Istituto di Biologia e Patologia Molecolari del CNR, Sapienza Università di Roma, Rome, Italy; 3 Dipartimento di Biologia e Biotecnologie “C. Darwin”, Sapienza Università di Roma, Rome, Italy; 4 Department of Biomedical Sciences, University of Padova, via Marzolo 3, 35131 Padua, Italy; 5 Department of Pathophysiology and Transplantation (DePT), Dino Ferrari Centre, University of Milan, Neuroscience Section, IRCCS Foundation Ca’ Granda Ospedale Maggiore Policlinico, Via Francesco Sforza 35, 20122 Milan, Italy

**Keywords:** TDP-43, Skeletal muscles, Dlg, Neuromuscular junctions, ALS

## Abstract

**Background:**

The ribonuclear protein TDP-43 has been implicated in the pathophysiology of amyotrophic lateral sclerosis (ALS), with genetic mutations being linked to the neurological symptoms of the disease. Though alterations in the intracellular distribution of TDP-43 have been observed in skeletal muscles of patients suffering from ALS, it is not clear whether such modifications play an active role in the disease or merely represent an expression of muscle homeostatic mechanisms. Also, the molecular and metabolic pathways regulated by TDP-43 in the skeletal muscle remain largely unknown. Here, we analyze the function of TBPH, the *Drosophila melanogaster* ortholog of TDP-43, in skeletal muscles.

**Results:**

We modulated the activity of TDP-43 in Drosophila muscles by means of RNA interference and observed that it is required to promote the formation and growth of neuromuscular synapses. TDP-43 regulated the expression levels of Disc-large (Dlg), and restoring Dlg expression either in skeletal muscles or in motoneurons was sufficient to suppress the locomotive and synaptic defects of TDP-43-null flies. These results were validated by the observation of a decrease in Dlg levels in human neuroblastoma cells and iPSC-differentiated motoneurons derived from ALS patients, suggesting similar mechanisms may potentially be involved in the pathophysiology of the disease.

**Conclusions:**

Our results help to unveil the physiological role of TDP-43 in skeletal muscles as well as the mechanisms responsible for the autonomous and non-autonomous behavior of this protein concerning the organization of neuromuscular synapses.

## Background

Amyotrophic lateral sclerosis (ALS) is a devastating disease characterized by the progressive denervation of skeletal muscles followed by motoneuron degeneration and loss. Its pathology is related to biochemical defects and genetic mutations in the ribonuclear protein (RNP) TDP-43 which have been detected in the great majority of patients and are linked to the neurological symptoms of the disease [1, 2]. Histological studies of the nervous system of affected individuals have revealed the presence of protein inclusions consisting of misfolded, abnormally phosphorylated TDP-43 [3–5]. The pathological modifications affect the subcellular distribution of TDP-43, which is found throughout the cytoplasm instead of its normal nuclear localization. Even though the TDP-43 alterations described above were mostly identified in the upper and lower motoneurons, additional brain regions and cell types were recently implicated in the pathology of ALS. A critical role for glia and immune cells has been recognized in the progression of the disease which has been confirmed in different animal models [6–10]. In this respect, we have reported that the endogenous TDP-43 (TBPH) is localized in glial cells in *Drosophila melanogaster*, where it prevents motoneuron degeneration. This supports the idea that ALS may have a non-neuronal origin [11]. In consonance with this hypothesis, pathological modifications of TDP-43 have been found in the skeletal muscles of patients with different neuromuscular diseases such as inclusion body myositis (IBM) and sporadic, familial forms of ALS [12]. Likewise, overexpression of TDP-43 leads to age-related muscle weakness and degeneration in mice [13], zebrafish [14], and Drosophila [15, 16], suggesting that the regulation of TDP-43 function could play an important role in muscle physiology. A requirement for TDP-43 has also been reported for the in vitro differentiation of C2C12 myoblasts as well as for the regeneration of tibial muscles in vivo [17]. Notwithstanding these findings, the molecules and metabolic pathways regulated by TDP-43 in skeletal muscles remain largely unknown. It is not known either whether primary defects of TDP-43 activity at the muscle level could somehow trigger the degenerative course of ALS nor which could be its potential consequences regarding the formation and maintenance of neuromuscular synapses. In order to address these questions, we have analyzed the endogenous function of TBPH, the TDP-43 ortholog gene, in skeletal muscles of the fruit fly *Drosophila melanogaster.*

## Methods

### Fly strains

The following genotypes were used:

w[1118] - w[1118]; TBPH^Δ23^/Cyo-GFP [18] - w[1118]; TBPH^Δ142^/Cyo-GFP [18] - w[1118]; Mef2-GAL4/TM3-Sb - w[1118]; MHC-GAL4/TM3-Sb - w[1118]; UAS-mCD8::GFP/Cyo - w[1118]; UAS-TBPH/Cyo - w[1118]; UAS-TBPH^F/L^/TM3-Sb - w[1118]; UAS-hTDP/TM3-Sb - w[1118]; UAS-DLG(egfp)/UAS-DLG(egfp) - w[1118]; UAS-TBPH RNAi (#ID38377) [18] - w[1118]; UAS-GFP RNAi (#9330) - w[1118]; UAS-GFP RNAi (#9331) - w[1118], Dicer(X).

### Larval movement

Wandering 3rd instar larvae (about 96 h old) were picked from tubes and washed in a drop of demineralized water. If necessary, they were selected against different markers such as tubby or Cyo-GFP and placed into 6-cm-diameter dishes, filled with 0.7% agar. A single larva at the time was transferred into a 10-cm-diameter dish, filled with 0.7% agar. After 30 s of adaption period, the number of peristaltic waves were counted for a period of 2 min. Tested larvae were subsequently transferred to a fresh fly tube to check them, both for hatching (after 4 days) and for correct genotype selection. Generally, 20–25 larvae per genotype were tested.

### Survival rate

One- to 2-day-old adult flies were collected from the fly tube of the experimental cross in a 1:1 proportion of female and male and transferred to a fresh fly tube and stored in the incubator under controlled conditions (suitable temperature and humidity, 12 h light and 12 h night). Every second day, flies were transferred into a fresh fly tube without anesthesia and the number of deaths was scored. Approximately 200 flies per genotype were tested.

### Climbing assay

One- to 2-day-old adult flies were collected from the fly tube of the experimental cross in a 1:1 proportion of female and male and transferred into a fresh fly tube and maintained in an incubator as previously described. The day of the setting of the experiment was counted as day 0. Flies were tested on days 4, 7, 14, and 21. A 50-ml glass cylinder was divided into three parts, as bottom, middle, and top (5 cm each part). Flies were carefully flipped into the cylinder from the fly tube without any anesthesia and gently dropped to the bottom. After 30 s of adaptation period, flies were dropped again onto the bottom of the cylinder, and after the time interval of 15 s, the numbers of flies present in each part of the cylinder were scored. For each genotype, 3 trials per tube were done and the average of the scored fly numbers was considered as the final score. A minimum of 200 flies was tested for each genotype.

### Walking assay

Young flies 2–3 days old were tested for walking ability. A 145-mm dish was used. The bottom surface was divided in a grid of 1 cm × 1 cm squares to facilitate the measuring of the distance walked by flies. The fly without any anesthesia was placed in the middle of the dish, and after 30 s of adaptation to the environment, the distance walked by the fly was recorded for 30 s, counting the number of squares. A minimum of 50 flies were individually tested for each genotype.

### Immunohistochemistry

Wandering 3rd instar larvae were picked from the fly tube, in a drop of demineralized water, selected for the genotype, and maintained during the time of dissection in a 6-cm-diameter dish filled with 0.7% agarose dissolved in water. Individually picked larva was dissected on Sylgard plates, in Dissection Solution (128 mM NaCl, 2 mM KCl, 4 mM MgCl_2_, 0.1 mM CaCl_2_, 35.5 mM sucrose, and 5 mM Hepes (pH 7.2)). Larvae were pinned at both ends with minute pins (Austerlic Isect Pins 0.1 mm diameter, Fine Science Tools, Germany) and opened on the dorsal site with Spring scissors (Fine Science Tools, Germany). Once larva was opened, internal organs were removed and the interior was extensively washed with Dissection Solution leaving the muscle wall opened, pinned flat on the surface. The subsequent step was a fixation, generally done with 4% PFA in PBS for 20 min; however, in the case of glutamate receptors staining a methanol fixation of 5 min at − 20 °C was performed. Fixation solution was removed with 3 washes in PBS-T (PBS 1× supplemented with 0.1% (v/v) Tween20) for 5 min each. After a blocking step of 30 min in blocking solution (5% NGS (normal goat serum (#S-1000 Chemicon) in PBS-T buffer), larvae were incubated overnight at 4 °C in primary antibodies diluted in blocking solution. The day after, primary antibody was removed with three washes of 10 min each with PBS-T and a further blocking step of 30 min was performed before secondary antibody addition. All secondary antibodies were diluted in blocking solution. An incubation was 2 h long, carried out at the room temperature. Excess of antibody was removed by 3 subsequent washes of 20 min each in PBS-T. Finally, dissected-stained larvae were incubated overnight at 4 °C in Slowfade®Gold antifade (#S36936 Life Technologies) reagent, before being mounted on a glass slide: anti-HRP (#323-005-021, lot:104838, Jackson 1:150), anti-GFP (#A11122, lot:1789911, Life Technologies 1:200), anti-GluRIIA 8B4D2c (DSHB 1:15), anti-Dlg 4F3c (DSHB 1:250), anti-Futsch 22C10s (DSHB 1:50), Alexa-Fluor® 488 (mouse #A11001 or rabbit #A11008 1:500), and Alexa-Fluor® 555 (mouse #A21422 or rabbit #A21428 1:500).

### Acquisition and quantification of confocal images

In each experiment, the genotypes of interest were processed simultaneously, and the images were acquired using the same settings. Images of muscles 6 and 7 on second abdominal segment were acquired using the LSM Zeiss Software on a Zeiss 510Meta confocal microscope (63 × oil lens) and then analyzed using ImageJ (Wayne Rasband, NIH). For the quantification of pre- and postsynaptic markers, samples were double labeled with anti-HRP and the marker of interest: the ratio between the mean intensity of the marker and the HRP was calculated for each bouton of the terminal [18, 19].

### Quantification of boutons

Boutons were stained with an anti-HRP antibody. The shape of boutons was evaluated as regular if they were round and with a smooth surface, with an equal diameter on both axes. On the other hand, boutons were considered as irregular if the shape was not round, the membrane was wrinkled, and the diameter of one axe was different compared to the other one [19].

### Electrophysiology on NMJ of the third instar larva preparation

Larval body wall preparations were dissected out in Ca2+-free HL3 solution from third instar larvae pinned on Sylgard-coated petri dishes. The central nervous system was excised by cutting segmental nerve roots. After replacing Ca2+-free HL3 solution with Ca2+ 1 mM HL3, postsynaptic potentials at neuromuscular junction of fiber 6/7 of abdominal segments A3/A4 were intracellularly recorded, at room temperature in current-clamp condition, using an intracellular microelectrode (tip diameter 0.5 μm, 15 MΩ resistance). The recorded signal was amplified by a current-clamp amplifier (SEC 05, NPI, Tamm, Germany), digitized at 10-kHz sampling rate using an A/D interface (National Instruments, Austin, TX, USA) and fed to a computer for display and storage using an appropriate software (Win EDR, Strathclyde University, Glasgow, UK).

Fibers with a resting membrane potential below − 60 mV were considered for the experiment. In these fibers, membrane potential was set at − 70 mV throughout the experiment by injecting current through the intracellular electrode. Evoked postsynaptic potentials (EPSPs or excitatory junctional potentials or EJPs) were recorded by stimulating at 0.1 Hz (pulse duration 0.4 ms; 1.5 threshold voltage) the segmental nerve using a suction electrode (tip diameter ~ 10 μm) connected to a stimulator (S88, Grass, Pleasanton, CA, USA) through a stimulus isolation unit (SIU5, Grass, Pleasanton, CA, USA). Intracellular recordings were analyzed offline using pClamp software (pClamp, Axon, Sunnyvale, CA, USA). Statistical comparisons and graphs were made using Graphpad software (Graphpad, La Jolla, CA, USA) or MATLAB (Matworks, Natick, MA, USA).

### Immunoprecipitation

Protein G magnetic beads (#10003D Invitrogen) were washed two times with PBS + 0.02% Tween and coated with anti-FLAG M2 monoclonal antibody (#F3165, Lot:SLBQ7119V, Sigma). Thoraces or heads of adult flies were cut and stored in lysis buffer containing 20 mM Hepes, 150 mM NaCl, 0.5 mM EDTA, 10% Glycerol, 0.1% Triton X-100, 1 mM DTT, and protease inhibitor (#04 693 159 001 Roche). Samples were homogenized with a Dounce homogenizer, and major debris were removed by centrifugation step of 5 min at 0.4*g* at 8 °C. The pretreated beads and tissue extracts were mixed and incubated for 30 min at 4 °C. After this binding step, beads were washed five times with washing buffer (20 mM Hepes, 150 mM NaCl, 0.5 mM EDTA, 10% glycerol, 0.1% Triton X-100, 1 mM DTT, protease inhibitor, 0.2% DOC, 0.5 M Urea) using DynaMagTM-Spin (#123.20D Invitrogen). RNA transcripts bound by Flag-tagged TBPH were extracted treating the beads with Trizol (#15596026 Ambion), and RNA was precipitated with isopropanol adding glycogen (#R0551 Thermo Scientific). Retro-transcription was performed with Superscript III First-Strand Synthesis (#18080-093 Invitrogen) and oligo-dT and subjected to real-time PCR with gene-specific primers, whose sequences are listed below.

**Table:** 

Target	fw primer	rv primer
**Rpl11**	5′-CCATCGGTATCTATGGTCTGGA-3′	5′-CATCGTATTTCTGCTGGAACCA-3′
**Syntaxin**	5′-TGTTCACGCAGGGCATCATC-3′	5′-GCCGTCTGCACATAGTCCATAG-3′
**Hdac-6**	5′-CGAGCGGCTGAAGGAGAC-3′	5′-ACCAGATGGTCCACCAATTCG-3′
**Dlg**	5′-ACTGGGCTTCTCAATTGCCG-3′	5′-CCAGTTCGTGCGTTACGTTC-3′

In order to calculate the enrichment fold, initially, all data were normalized to the respective inputs. The signal was represented by how many more fold increase was measured compared to the control signal. The enrichment was calculated according to the 2^-ΔΔCt^ method.

The results were derived from three independent immunoprecipitation experiments [19, 20].

### Protein extraction

To collect adult heads, flies were flash-frozen in liquid nitrogen for 10 s and immediately vortexed to easily detach heads from bodies. Heads were subsequently transferred into Lysis buffer (150 mM Tris, 5 mM EDTA, 10% glycerol, 5 mM EGTA, 50 mM NaF, 4 M urea, 5 mM DTT, and protease inhibitors (#04 693 159 001 Roche)). After a squeezing step, performed both, manually and mechanically, the homogenized samples were gotten rid of major debris by centrifugation at 0.5×*g* for 6 min on 4 °C. The protein concentration of the collected supernatant was quantified with Quant-iT™ Protein Assay Kit (#Q33212 Invitrogen), following the supplier protocol.

Transfected neuroblastoma cell line SH-SY-5Y was resuspended in iced RIPA buffer added of protease inhibitors (#04693159001 Roche) and subjected to sonication (Biorupture sonication system, Diagenode).

Lysates were quantified (BCA Protein kit #23225 Thermo Scientific), following the supplier protocol.

### RNA extraction and qRT-PCR

RNA was extracted from Drosophila adult heads, 1 day aged and sex-matched, of both wildtype and TBPH-null alleles (*tbph*^D23^ and *tbph*^D142^) and from human MN differentiated cells with RNeasy Microarray tissue kit (QIAGEN #73304) and treated with Turbo DNA-free kit (Ambion #AM1907).

Retro-transcription was performed with Superscript III First-Strand Synthesis (#18080-093 Invitrogen) using oligo-dT with the exception of the analysis of the Drosophila intronic region for which random hexamers have been used. Real-time PCR was carried out with the primers listed below using Platinum Syber Green (#11733-038 Thermo Fisher) on a Bio-Rad CFX96 qPCR System, and minus-retro control has been performed. The primer sets used are listed in the below table; Sdha and GAPDH genes have been used as Drosophila and human reference, respectively.

**Table:** 

Target	fw primer	rv primer
**Drosophila**		
**d-DLG exon 1–4**	5′-CTGTGCCAAACGCATGTCC-3′	5′-TTCACAGCTCAATGGCTCCT-3′
**d-DLG intron 1–4**	5′-TCTGCGACCCAGTTTTCCAC-3′	5′-TCGCCCGTGGTTGTAATTGT-3′
**Sdha**	5′-CATGCTGCTGTGTTCCGCGA-3′	5′-ACCATCCAGGGGCTTGCTGA-3′
**Human**		
**h-DLG**	5′-GTTGCACAATATCGACCTGAAGA-3′	5′-GGGATCGCTTCTGGCTAGTTC-3′
**GAPDH**	5′-CTGGGCTACACTGAGCACC-3′	5′-AAGTGGTCGTTGAGGGCAATG-3′

### SDS-PAGE

Protein samples were diluted in 1x Laemmli buffer (composition of 5x: 0.3 M Tris-HCl pH 6.8, 50% glycerol, 10% SDS, 25% β-mercaptoethanol, 0.05% bromophenol blue) to reach the same concentration among all and then boiled at 95 °C for 5 min. Afterwards, they were loaded on a polyacrylamide gel.

The loaded gel was placed into a chamber with 1× running buffer (10× running buffer: 30.28 g Tris, 114.13 g glycine, 10 g SDS in 1 l water). The conditions set were 25 mA per gel.

### Western blot

When proteins were separated by the electrophoresis, they were transferred to a nitrocellulose membrane Amersham™ Protran™ 0.2 μm NC (Life Science). The western blot sandwich was put into the chamber, filled with transfer buffer 1× containing 20% methanol (transfer buffer 10×: 30 g Tris, 144 g glycine in 1 l water). The transfer lasted 1 h at 350 mA. The membrane was incubated with a solution of 5% milk in 1× TBS 0.01% Tween (TBS-T) for 30 min at a room temperature on a shaker (TBS buffer 10×: 24.2 g Tris, 80 g NaCl in 1 l water, pH 7.6). After blocking, the membrane was set into dilution of primary antibody with TBS-T with 5% milk. It was placed at 4 °C overnight. When the incubation with primary antibody was over, five washes with TBS-T followed, 5 min each. Next, the membrane was incubated with the secondary antibody diluted in TBS-T with 5% milk for 1 h at room temperature. The protein detection was performed with SuperSignal®West Femto Maximum Sensitivity Substrate (#TB260893 Thermo Fisher Scientific). Primary antibodies include anti-Dlg 4F3c (DSHB 1:10000), anti-TBPH (home-made 1:4000), anti-tubulin (#CP06, Lot:2681308, Calbiochem 1:4000), anti-TDP-43 (#12892-1-AP, Lot:00016371, Proteintech 1:4000), anti-Dlg 2D11 (#SC9961, Lot:G2617, Santa Cruz 1:1000), and anti-GAPDH (#SC25778, Lot:G1008, Santa Cruz 1:2000). Secondary antibodies include anti-mouse-HRP (#31430 Thermo Fisher Scientific 1:30000(flies), 1:10000(cells)) and anti-rabbit-HRP (#31460 Thermo Fisher Scientific 1:10000).

### Cell culture and RNA interference

SH-SY-5Y neuroblastoma cell line was cultured in standard conditions in DMEM-Glutamax (#31966-021, Thermo Fisher Scientific) supplemented with 10% fetal bovine serum and 1 × antibiotic-antimycotic solution (#A5955; Sigma). RNA interference of TDP-43 was achieved using HiPerfect Transfection Reagent (#301705, Qiagen) and siRNA specific for human TDP43 (5′-gcaaagccaagaugagccu-3′); as control, siRNA for luciferase was used (5′-uaaggcuaugaagagauac-3′; Sigma). Immediately before transfection, 2–4 × 10^5^ cells were seeded in 6-well plates in 1.4 ml of medium containing 10% fetal serum. A volume of 3 μl of each siRNA (40 μM solution in water) was added to 91 μl of Opti-MEM I reduced serum medium (#51985-026, Thermo Fisher Scientific) and incubated 5 min at room temperature, and subsequently, 6 μl of HiPerfect Transfection Reagent was added. The silencing procedure was performed again after 24 and 48 h.

### Human iPSC culture and MN differentiation

Human iPSC culture and MN differentiation were already described in [21]. All the studies performed with human samples were in compliance with the Code of Ethics of the World Medical association (Declaration of Helsinki) and with the national legislation and institutional guidelines. Briefly, fibroblasts from dermal biopsies (Eurobiobank) from ALS patients (TDP-43 mutations #1:G294V; #2:G378S) and controls were reprogrammed into iPSCs with CytoTune-iPS 2.0 Sendai reprogramming Kit (#A16517, Thermo Fisher) and differentiated into MNs with the multistep protocol described by Ng [22].

### Statistical analysis

All statistical analysis was performed with Prism (GraphPad, USA) version 5.1. One-way ANOVA with Bonferroni correction and *t* test with Mann-Whitney correction were applied as statistical test. In all figures, all the values were presented as the mean and the standard error of the mean (SEM). Statistical significance was portrayed as *p < 0.05, **p < 0.01, ***p < 0.001, and ****p < 0.0001.

## Results

### The suppression of TBPH in muscles affects Drosophila locomotion and life span

**Fig. 1:**
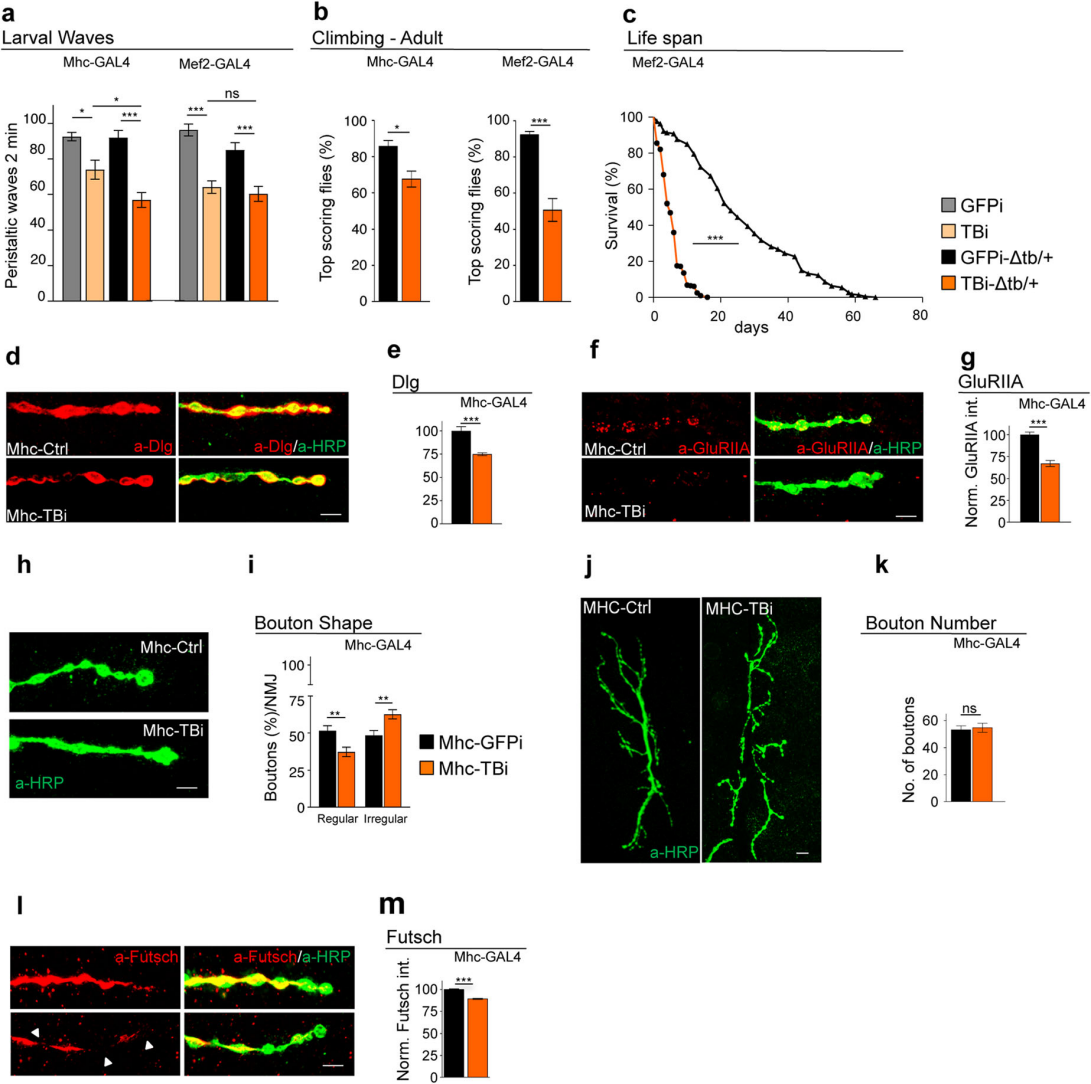
Suppression of TBPH in muscle affects locomotion and life span. **a** Number of peristaltic waves of Ctrl (+/+;driverGAL4/UAS-GFP-IR), TBi (+/+;driverGAL4/UAS-TBPH-IR), Ctrl-Δtb/+ (tbph^Δ23^/ +;driverGAL4/UAS-GFP-IR), and TBi-Δtb/+ (tbph^Δ23^/+;driverGAL4/UAS-TBPH-IR) using MHC-GAL4 (left panel) and Mef2-GAL4 (right panel). *n* = 20. **b** Climbing assay of Ctrl-Δtb/+ and TBi-Δtb/+ using MHC-GAL4 (left panel) and Mef2-GAL4 (right panel) at day 7. *n* = 200. **c** Life span analysis of TBi-Δtb/+ compared to Ctrl-Δtb/+ using Mef2-GAL4. *n* = 200. **d** Confocal images of the third instar NMJ terminals in muscle 6/7 second segment stained with anti-HRP (in green) and anti-Dlg (in red) in MHC-Ctrl (tbph^Δ23^/+;MHC-GAL4/UAS-GFP-IR) and MHC-TBi (tbph^Δ23^/+;MHC-GAL4/UAS-TBPH-IR). **e** Quantification of Dlg intensity normalized on Ctrl. *n* > 200 boutons. **f** Confocal images of the third instar NMJ terminals in muscle 6/7 second segment stained with anti-HRP (in green) and anti-GluRIIA (in red) in MHC-Ctrl and MHC-TBi. **g** Quantification of GluRIIA intensity normalized on Ctrl. *n* > 200 boutons. **h** Confocal images of the third instar NMJ terminals in muscle 6/7, second segment stained with anti-HRP (in green) in MHC-Ctrl and MHC-TBi. The details of the bouton shape are shown. **i** Quantification of the bouton shape. *n* = 200. **j** Confocal images of third instar NMJ terminals in muscle 6/7 second segment stained with anti-HRP (in green) in MHC-Ctrl and MHC-TBi. **k** Quantification of bouton number. *n* = 15. **l** Confocal images of third instar NMJ terminals in muscle 6/7 second segment stained with anti-HRP (in green) and anti-Futsch (in red) in MHC-Ctrl and MHC-TBi. **m** Quantification of Futsch intensity normalized on Ctrl. *n* > 200 boutons. ns not significant, *p < 0.05, **p < 0.01, ***p < 0.001 calculated by one-way ANOVA (for more than two groups) and *t* test (for two groups) and log rank test (for survival analysis) error bars SEM. Scale bar 10 μm (panel **j**) and 5 μm (in panels **d**, **f**, **h**, and **l**)

We have previously described that TBPH is expressed in Drosophila muscles, being present in myocytes from larval stages until adulthood [16]. To analyze its role in these tissues, we suppressed TBPH expression by means of RNA interference (RNAi). Two different GAL4 lines were utilized: *Mhc* (myosin heavy chain)-GAL4, with predominant expression in larval muscles, and *Mef2*-GAL4, expressed during muscle development and in adulthood [23]. The expression of anti-TBPH RNAi (TBi) in either *Mhc*-GAL4 or *Mef2*-GAL4 significantly affected the locomotor capacities of flies at both larval and adult stages (Fig. 1a, b; Additional file 1 Fig.S1). Moreover, the life span of *Mef2*-GAL4 flies expressing TBi was strongly compromised compared with that of *Mef2*-GAL4 control flies expressing GFP-RNAi (GFPi) (Fig. 1c). It could also be inferred that a reduction of one copy of the endogenous TBPH gene due to the expression of TBi results in an exacerbation of locomotive defects (*Mhc*-GAL4, TBPH^−^/_+_, TBi vs *Mhc*-GAL4, TBPH^−^/_+_, GFP-RNAi). This was an indication that these phenotypes are gene dose-sensitive and rather specific (Fig. 1a). At the cellular level, the expression of TBi strongly affected the organization of neuromuscular synapses, as revealed by the localization of postsynaptic proteins in muscle cell membranes (Fig. 1d–g). More specifically, we detected strongly reduced levels of the postsynaptic protein Disc-large (Dlg) in TBPH-RNAi-treated muscles compared with controls and observed the presence of numerous gaps of a pathological nature in the distribution of Dlg around motoneuron axons (Fig. 1d, e). Furthermore, the postsynaptic distribution and clustering of glutamate receptors (GluRIIA) was found to be impaired in RNAi-treated flies relative to controls, which indicates that TBPH muscle functionality is a requirement to prevent postsynaptic membrane disorganization (Fig. 1f, g). The downregulation of TBPH in Drosophila muscles also provoked non-autonomous alterations in the structure of presynaptic terminals, which showed a loss of the characteristics round and smooth shape of the synaptic boutons (Fig. 1h, i) without affecting their total number (Fig. 1j, k). At a molecular level, these morphological alterations were associated with a significative reduction in the levels of the presynaptic microtubule binding protein *futsch*, homolog to the human protein MAP1B, involved in the organization of the synaptic microtubule cytoskeleton (Fig. 1l, m).

### The role of TBPH in muscles is sufficient to promote neuromuscular synapse growth and innervation

**Fig. 2:**
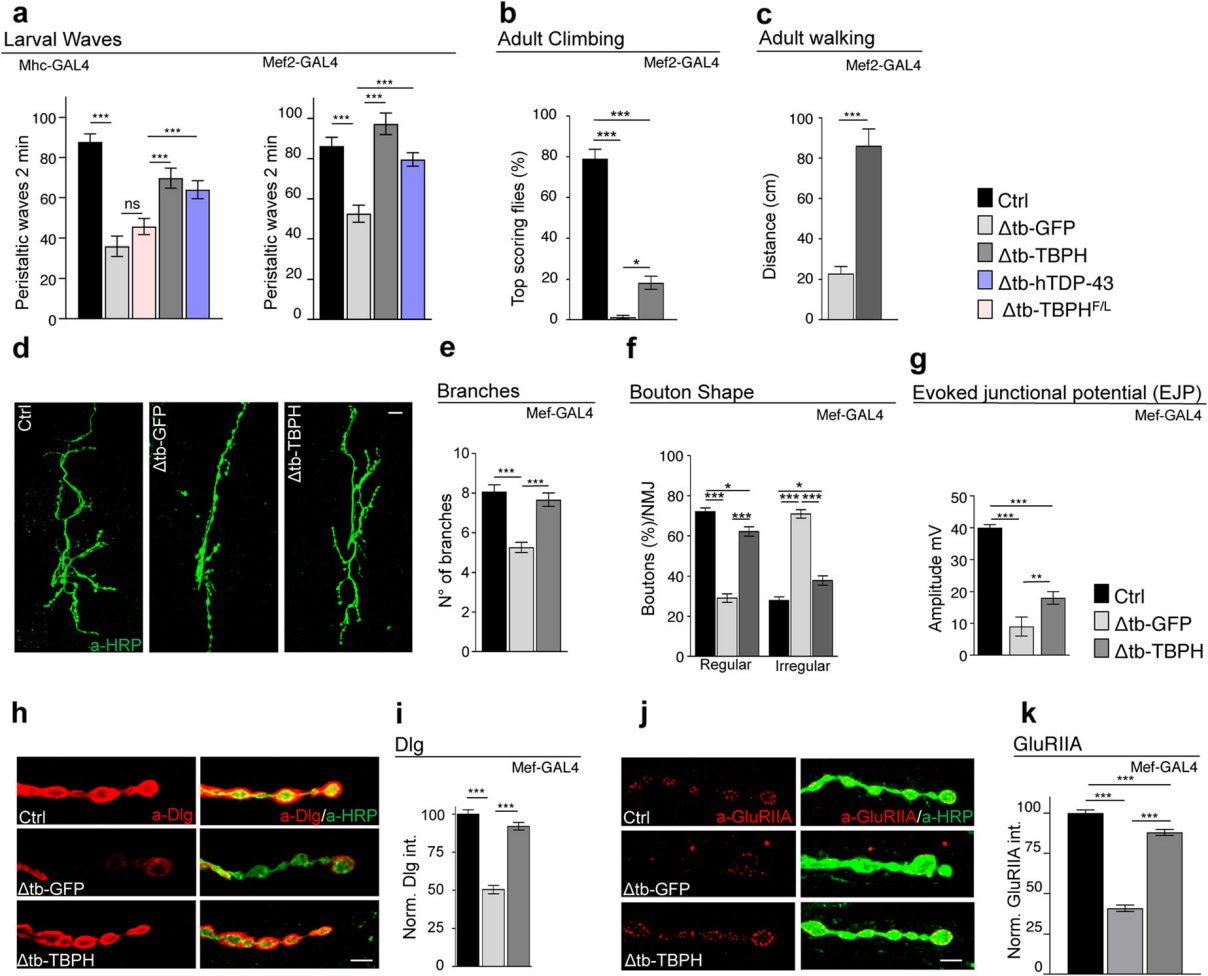
The muscular function of TBPH is sufficient to promote neuromuscular synaptic growth. **a** Number of peristaltic waves of Ctrl (*w*^1118^), Δtb-GFP (tbph^Δ23^/tbph^Δ23^;driver-GAL4/UAS-GFP), Δtb-TBPH (tbph^Δ23^,UAS-TBPH/tbph^Δ23^;driver-GAL4/+), Δtb-hTDP-43 (tbph^Δ23^/tbph^Δ23^;driver-GAL4/UAS-TDP-43), and Δtb-TBPH^F/L^ (tbph^Δ23^/tbph^Δ23^;driver-GAL4/UAS-TBPH^F/L^) using Mhc-GAL4 (left panel) and Mef2-GAL4 (right panel). *n* = 20. **b** Climbing assay of Ctrl, Δtb-GFP, and Δtb-TBPH using Mef2-GAL4 at day 2. *n* = 200. **c** Walking assay analysis of Δtb-GFP and Δtb-TBPH using Mef2-GAL4 at day 2. *n* = 100. **d** Confocal images of third instar NMJ terminals in muscle 6/7 second segment stained with anti-HRP (in green) in Ctrl, Δtb-GFP, and Δtb-TBPH. **e** Quantification of branch number. *n* = 15. **f** Quantification of the bouton shape. *n* = 200. **g** Evoked neurotransmitter release. Representative EJPs evoked by segmental nerve stimulation of Ctrl, Δtb-GFP, and Δtb-TBPH in muscle fiber 6/7 of A3 in third instar larvae. For each fiber, 15 EPPs following 0.5-Hz stimulation were considered. **h** Confocal images of third instar NMJ terminals in muscle 6/7 second segment stained with anti-HRP (in green) and anti-Dlg (in red) in Ctrl, Δtb-GFP, and Δtb-TBPH. **i** Quantification of Dlg intensity normalized on Ctrl. *n* > 200 boutons. **j** Confocal images of third instar NMJ terminals in muscle 6/7 second segment stained with anti-HRP (in green) and anti-GluRIIA (in red) in Ctrl, Δtb-GFP, and Δtb-TBPH. **k** Quantification of GluRIIA intensity normalized on Ctrl. *n* > 200 boutons. *p < 0.05, **p < 0.01, and ***p < 0.001 calculated by one-way ANOVA, error bars SEM. Scale bar 10 μm (in **d**) and 5 μm (in **h** and **j**)

In order to further characterize the function of TBPH in skeletal muscles, we studied the consequences of the presence of TBPH in TBPH-null backgrounds (Δtb-TBPH) using the drivers *Mhc*-GAL4 or *Mef2*-GAL4. TBPH expression was found to result in the significative recovery of the normal locomotive behavior of larvae and adult flies (Fig. 2a–c and Additional file 2 Fig. S2a). We also found that the muscle expression of human TDP-43 in a TBPH-null background (Δtb-hTDP-43) similarly led to a motility recovery, suggesting that the role of TDP-43/TBPH is conserved in skeletal muscles (Fig. 2a and Additional file 2 Fig. S2b). On the contrary, the expression of a RNA-binding-deficient isoform of TBPH (TBPH^F/L^) was not able to revert TBPH-minus phenotypes, demonstrating that RNA binding is essential for TDP-43/TBPH functionality in these tissues (Fig. 2a) [19]. The TBPH-induced recovery of muscle function stimulated the growth of motoneuron axons as well as the formation of terminal branches and new synaptic boutons in TBPH-minus flies (Fig. 2d–f and Additional file 2 Fig. S2 c and d). Interestingly, the non-autonomous rescue of presynaptic terminals was followed by the reestablishment of evoked junction potentials (EJPs) between motoneurons and their underlying muscles, suggesting the recovery of synaptic transmission in TBPH-expressing muscles compared with controls (Fig. 2g).

### TBPH promotes the assembly of neuromuscular synapses by regulating muscle and neuronal levels of Dlg

**Fig. 3:**
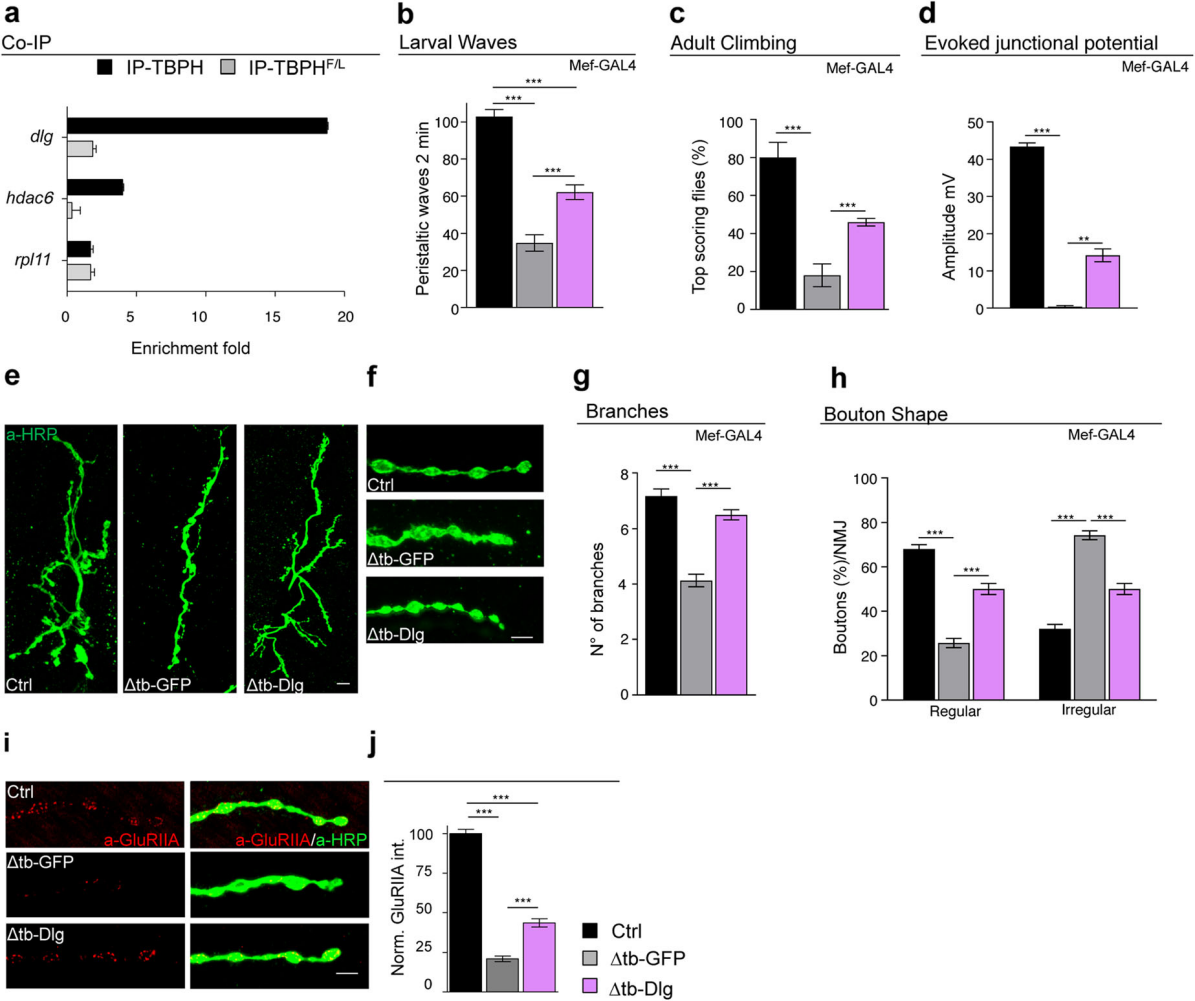
TBPH in the muscle promotes synaptic growth through the regulation of Dlg levels. **a** qRT-PCR analysis of mRNAs immunoprecipitated by Flag-tagged TBPH (UAS-TBPH/+;Mef2-GAL4/+, IP-TBPH) and its mutant variants TBPH^F/L^ (+/+;UAS-TBPH ^F/L^/Mef2-GAL4, IP-TBPH^F/L^) in adult thoraxes. The *dlg* enrichment folds was referred to *rpl-11* (negative control); *hdac6* has been used as the positive control. *n* = 3 (biological replicates). Individual data values are provided in Additional file 4. Individual Data Values.xls. **b** Number of peristaltic waves of Ctrl (*w*^1118^), Δtb-GFP (tbph^Δ23^/tbph^Δ23^;Mef2-GAL4/UAS-GFP), and Δtb-Dlg (tbph^Δ23^,UAS-Dlg/tbph^Δ23^;Mef2-GAL4/+). *n* = 20. **c** Climbing assay of Ctrl, Δtb-GFP, and Δtb-Dlg using Mef2-GAL4 at day 2. *n* = 200. **d** Evoked neurotransmitter release. Representative EJPs evoked by segmental nerve stimulation of Ctrl, Δtb-GFP, and Δtb-Dlg in muscle fiber 6/7 of A3 in third instar larvae. For each fiber, 15 EPPs following 0.5-Hz stimulation were considered. **e** Confocal images of third instar NMJ terminals in muscle 6/7 second segment stained with anti-HRP (in green) in Ctrl, Δtb-GFP, and Δtb-Dlg. **f** Confocal images of third instar NMJ terminal boutons in muscle 6/7 second segment stained with anti-HRP (in green) in Ctrl, Δtb-GFP, and Δtb-Dlg. **g** Quantification of branch number. *n* = 15. **h** Quantification of the bouton shape. *n* = 200. **i** Confocal images of third instar NMJ terminals in muscle 6/7 second segment stained with anti-HRP (in green) and anti-GluRIIA (in red) in Ctrl, Δtb-GFP, and Δtb-Dlg. **j** Quantification of GluRIIA intensity normalized on Ctrl. *n* > 200 boutons. **p < 0.01 and ***p < 0.001 calculated by one-way ANOVA, error bars SEM. Scale bar 10 μm (in **e**) and 5 μm (in **f** and **i**)

**Fig. 4:**
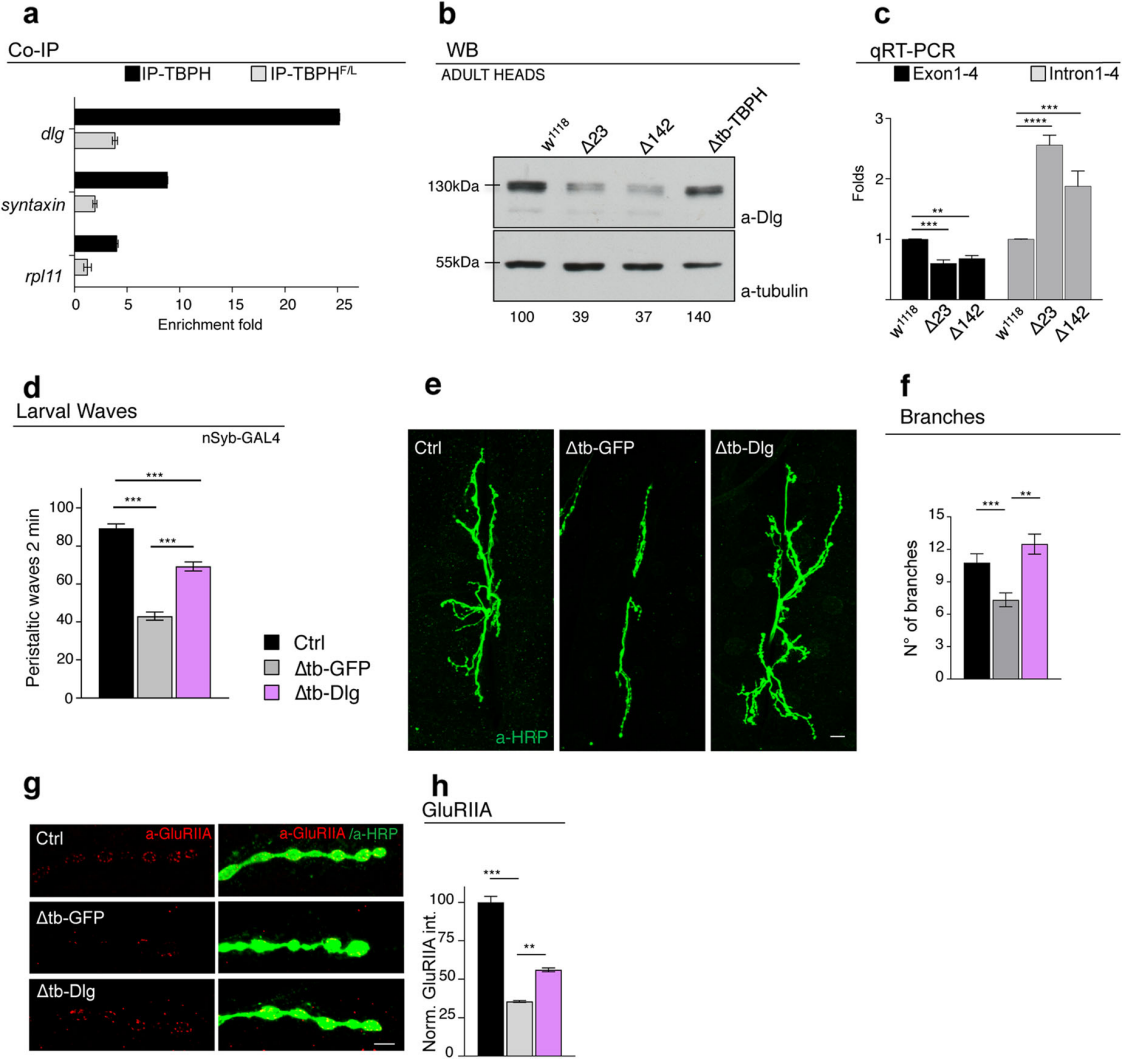
TBPH in neurons promotes synaptic growth through the regulation of Dlg levels. **a** qRT-PCR analysis of mRNAs immunoprecipitated by Flag-tagged TBPH (Elav-GAL4/UAS-TBPH/+;+/+, IP-TBPH) and its mutant variants TBPH^F/L^ (Elav-GAL4/+;UAS-TBPH ^F/L^/+, IP-TBPH^F/L^) in adult heads. The *dlg* enrichment folds was referred to *rpl-11* (negative control); *syntaxin* has been used as the positive control. *n* = 3 (biological replicates). Individual data values are provided in the supplementary file data values. Individual data values are provided in Additional file 4. Individual Data Values.xls. **b** Western blot analysis of lane 1 (*w*^1118^), lane 2 (tbph^Δ23^/tbph^Δ23^), lane 3 (tbph^Δ142^/tbph^Δ142^), and lane 4 (tbph^Δ23^,Elav-GAL4/tbph^Δ23^,UAS-TBPH). Adult brains, 1 day old, were probed with anti-Dlg antibody and anti-alpha-tubulin as loading control. Quantification of normalized amounts was reported below each lane. *n* = 3 (biological replicates). Individual data values are provided in the supplementary file data values. **c** qRT-PCR analysis of DLG. mRNA from adult heads of the Ctrl (*w*^1118^) and the two null alleles of TBPH (tbph^Δ23^/tbph^Δ23^ and tbph^Δ142^/tbph^Δ142^) were retrotranscribed with oligo-dT and random hexamers for exons 1–4 and introns 1–4, respectively; DLG transcripts were normalized on *Sdha* reference gene, and folds were calculated versus wildtype control. *n* = 2 (biological replicates; real-time reactions performed in triplicate for each biological replicate). Individual data values are provided in the supplementary file individual data values. **p < 0.01, ***p < 0.001, and ****p < 0.0001 calculated by one-way ANOVA, error bars SEM. **d** Number of peristaltic waves of Ctrl (*w*^1118^), Δtb-GFP (tbph^Δ23^/tbph^Δ23^;nSyb-GAL4/UAS-GFP), and Δtb-Dlg (tbph^Δ23^,UAS-Dlg/tbph^Δ23^;nSyb-GAL4/). *n* = 20. **e** Confocal images of third instar NMJ terminals in muscle 6/7 second segment stained with anti-HRP (in green) in Ctrl, Δtb-GFP, and Δtb-Dlg. **f** Quantification of branch number. *n* = 15. **g** Confocal images of third instar NMJ terminals in muscle 6/7 second segment stained with anti-HRP (in green) and anti-GluRIIA (in red) in Ctrl, Δtb-GFP, and Δtb-Dlg. **h** Quantification of GluRIIA intensity normalized on Ctrl. *n* > 200 boutons. **p < 0.01 and ***p < 0.001 calculated by one-way ANOVA, error bars SEM. Scale bar 10 μm (in **e**) and 5 μm (in **g**)

The expression of TBPH in the muscles of TBPH-null flies also resulted in an almost complete restoration of the cytoplasmic levels and postsynaptic distribution of Dlg around presynaptic terminals (Fig. 2h, i and Additional file 2 Fig. S2e and f). This was complemented by a significative reorganization of glutamate receptors in well-defined clusters at the postsynaptic membrane level (Fig. 2j, k and Additional file 2 Fig. S2g and h). Regarding the molecular basis of the relationship between TBPH and Dlg, the fact that several putative TBPH binding sites are present along the mRNA sequence of Dlg is suggestive of physical interaction points (Additional file 3: Table 1 and 2 and [24–26]). To test this possibility, we performed RIP assays employing a tagged version of TBPH expressed in vivo in Drosophila muscles. We observed that TBPH, but not TBPH^F/L^, was able to pull down Dlg mRNA (Fig. 3a). In addition, the ectopic expression of Dlg in Drosophila muscles via *Mef2*-GAL4 was able to restore motility and climbing in TBPH-null flies (Fig. 3b, c) and to re-establish evoked junction potentials (EJPs) between motoneurons and their underlying muscles (Fig. 3d). Moreover, the postsynaptic expression of Dlg was observed to promote the non-autonomous growth of motoneuron axons (Fig. 3e–h) as well as the recovery of the postsynaptic organization of glutamate receptors at the neuromuscular membranes (Fig. 3i, j), indicating that Dlg is as a bona fide mediator of signaling pathways related to TBPH in Drosophila muscles. Dlg expression was in fact detected in motoneuron axons, which is consistent with a possible role of TBPH in the regulation of Dlg expression in neuronal tissues. In support of this idea, RIP assays showed that TBPH binds Dlg mRNA in Drosophila brains upon TBPH expression through the pan-neuronal driver *elav-*GAL4 (Fig. 4a). Moreover, we found that the protein levels of Dlg appeared downregulated in adult heads of Drosophila TBPH^Δ23^ and TBPH^Δ142^ homozygous alleles compared to wildtype controls and, importantly, the decreased Dlg protein levels were restored by the expression of endogenous TBPH demonstrating a degree of specificity in the results observed (Fig. 4b). Regarding the molecular mechanisms behind Dlg protein defects, we quantified by qRT-PCR the primary and mature transcripts of Dlg mRNA. For these experiments, we designed a set of primers able to amplify the initial coding exons 1 and 4 present in the most conserved and longest isoforms of Dlg mature mRNA. To detect the primary transcripts of Dlg mRNA, we selected primers directed to the intronic region between the exons described above. As a result, we found that Dlg mature mRNA was downregulated in TBPH-null alleles (Fig. 4c, left graph). On the contrary, the primary transcripts of Dlg appeared upregulated in mutant flies compared to wildtype controls (Fig. 4c, right graph) suggesting that TBPH function might be required to regulate the splicing dynamics of Dlg mRNA in vivo.

At a functional level, we determined that the presynaptic expression of Dlg in TBPH-minus flies promotes the recovery of the locomotive problems characteristic of TBPH-null larvae (Fig. 4d) and stimulates the presynaptic growth of motoneuron axons and the formation of new terminal branches and synaptic boutons (Fig. 4e, f). Additionally, the neuronal expression of Dlg induced the non-autonomous clustering of glutamate receptors in postsynaptic membranes of TBPH-minus NMJs (Fig. 4g, h), demonstrating that TBPH regulates the pre- and postsynaptic levels of Dlg and is required in muscles and neurons to promote the formation of NMJs.

### Alterations in Dlg levels are conserved in human cells and motoneurons from ALS patients carrying mutations in TDP-43

**Fig. 5:**
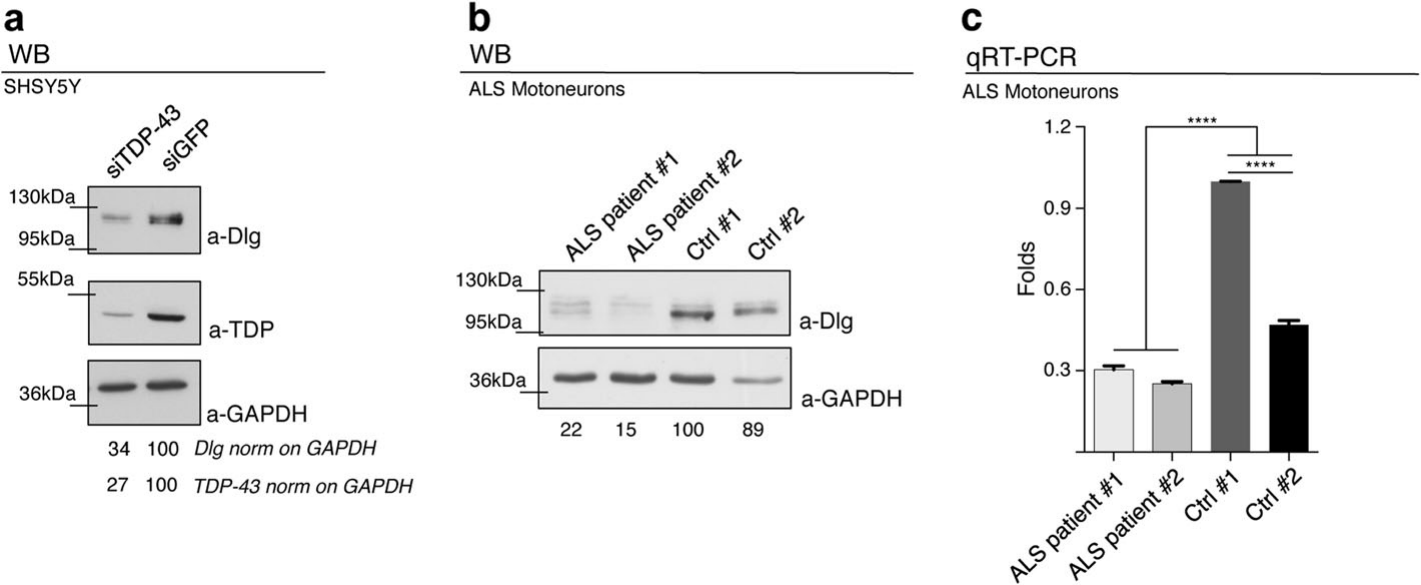
Dlg defects are conserved in human cells and motoneurons derived from ALS patients carrying TDP-43 mutations. **a** Western blot analysis on human neuroblastoma (SH-S5Y5) cell line probed for anti-Dlg, anti-GAPDH, and anti-TDP-43 in siGFP (GFP Ctrl) and siTDP-43 (TDP-43 silenced). The same membrane was probe with the three antibodies and the bands of interest were cropped. Quantification of normalized protein amount was reported below each lane, *n* = 3 (biological replicates). Individual data values are provided in Additional file 4. Individual Data Values.xls. **b** Western blot analysis probed for anti-Dlg and anti-GAPDH on human differentiated motoneurons derived from iPSCs of an ALS patient (ALS patient #1 and ALS patient #2) and a healthy control (Ctrl #1 and Ctrl #2). The same membrane was probe with the two antibodies, and the bands of interest were cropped. Quantification of normalized protein amount was reported below each lane, *n* = 3 (biological replicates). Individual data values are provided in Additional file 4. Data Values.xls. **c** qRT-PCR analysis of DLG. mRNA from human differentiated motoneurons were retrotranscribed with oligo-dT, and DLG transcripts were normalized on GAPDH (housekeeping) gene. *n* = 2 (biological replicates; real-time reactions performed in duplicate for each biological replicate). Individual data values are provided in the Additional file 4. Individual Data Values.xls. ****p < 0.0001 calculated by one-way ANOVA, error bars SEM

We explored whether changes in Dlg levels are associated to TDP-43 also in human cells. SH-SY5Y neuroblastoma cells in which TDP-43 expression had been greatly reduced by treatment with an anti-TDP-43 RNAi showed a concomitant, clear downregulation of the Dlg homolog protein SAP-97/DLG1 (Fig. 5a). Once established that regulatory mechanisms like those observed in Drosophila appear to be conserved in human tissues, we examined human DLG1 ortholog levels in motoneurons differentiated from iPS cell lines obtained from patients with mutations in TDP-43. Reductions in DLG1 protein and mRNA levels were detected in cells from ALS patients in comparison with those of healthy controls (Fig. 5b, c), suggesting that mechanisms analogous to those seen in Drosophila could also be operating in ALS.

## Discussion

Atrophy and weakness of body muscles are essential characteristics of ALS, mostly attributed to motoneuron degeneration. Experimental and patient studies have indicated that muscles likely play a primary role at the onset of the disease. We describe here that the suppression of TBPH at the muscle level results in locomotive defects, paralysis, and a life span reduction. At the molecular level, TBPH was found to be necessary to preserve the postsynaptic organization of neuromuscular junctions, assessed by the subcellular distribution of Dlg and GluRIIA on muscle membranes. No clear signs of muscle degeneration or atrophy were induced by expression of TBPH RNAi during larval development. In agreement with these results, we did not observe alterations in skeletal muscle development in TBPH-null alleles, suggesting that the role of TBPH in Drosophila muscles is restricted to the formation and differentiation of neuromuscular synapses [18, 19].

In consonance with the results described above, we observed that rescuing TBPH muscular function was sufficient to substantially restore the molecular organization of postsynaptic terminals as shown by the wildtype sharing of Dlg and GluRIIA. Moreover, the expression of TBPH in the muscles of TBPH-minus flies induced the non-autonomous growth of motoneurons axonal terminals as well as restored synaptic transmission, followed by a reversal to normality of locomotive behavior and life span. We also describe that the neurotrophic properties of TBPH depend on its RNA-binding capacity and are conserved in the human ortholog TDP-43, suggesting that similar phenomena likely occur in patients showing TDP-43 functional defects. In addition, TBPH was observed to regulate the expression levels of Dlg by interacting with its mRNA. Regarding the mechanisms behind these molecular interactions, we found that they are present in both skeletal muscles and motoneurons and might be required to regulate the splicing patterns of Dlg mRNA. In support of this idea, we showed that TBPH-minus flies presented reduced levels of mature Dlg mRNAs due to, most probably, defects in the processing of the immature transcripts.

Moreover, we observed that the more affected isoforms are the amplicons corresponding to the Dlg mRNA transcripts RB, RH, and RL that present higher homology with human DLG1 (63%, 61%, and 58%, respectively). Interestingly, these transcripts also display an increased number of coding exons and extended intronic regions enriched in TG repeats or TDP-43/TBPH putative RNA-binding sequences, reinforcing the notion that TBPH modulates Dlg protein expression through the molecular processing of the mRNA transcripts. In this direction, the genetic rescue of Dlg protein amounts in pre- or postsynaptic compartments was able to significantly restore the neurological defects caused by the absence of TBPH. Concerning the mechanism of such reversal, it has been described that Dlg is able to recruit adhesion molecules, scaffolding and signaling proteins to the plasma membranes through its PDZ domains [27, 28]. This would explain the autonomous and non-autonomous roles of TBPH in the formation of synaptic terminals [20, 29–31]. In this context, the expression of Dlg (regulated by TBPH/TDP-43) may promote the clustering of adhesion and signaling molecules like *fasciclin II* that can mediate trans-synaptic homophilic adhesions with the opposite synaptic membranes, contributing to the molecular assembly of the synapses [32]. Further experiments will be necessary, though, to verify these hypotheses.

In conclusion, our studies show that primary defects in TBPH function at the skeletal muscle level result in locomotive impairments and a reduction in the life span of Drosophila. The molecular mechanisms of these effects include the binding of TBPH to Dlg mRNA which leads to the regulation of Dlg expression levels in muscles and/or motoneurons. The latter is in turn associated to the assembly and functional organization of neuromuscular junctions. Crucially, we have observed that these mechanisms are also likely operative in human neural cell lines and motoneurons differentiated from ALS patients’ cells.

## Supplementary information

**Additional file 1: Figure S1.** Control of TBPH silencing. Western blot analysis on larval carcasses probed for anti-TBPH and anti-tubulin in tbph^Δ23^/+;Mef2-GAL4/UAS-GFP-IR and tbph^Δ23^/+;Mef2-GAL4/UAS-TBPH-IR. The same membrane was probed with the two antibodies and the bands of interest were cropped. *n* = 3 (biological replicates). Individual data values are provided in the Additional file 4. Individual Data Values.xls.

**Additional file 2: Figure S2. a.** Western blot analysis on larval carcasses probed for anti-TBPH and anti-tubulin in Ctrl (*w*^1118^), tbph^Δ23^/tbph^Δ23^, tbph^Δ23^,UAS-TBPH/tbph^Δ23^;Mef2-GAL4/+, tbph^Δ23^/tbph^Δ23^;Mef2-GAL4/UAS-TBPH^F/L^. The same membrane was probed with two antibodies and the bands of interest were cropped. *n* = 3 (biological replicates). **b.** Western blot analysis on larval carcasses probed for anti-TDP and anti-tubulin in Ctrl (*w*^1118^) and tbph^Δ23^/tbph^Δ23^;Mef2-GAL4/UAS-TDP-43 The same membrane was probed with two antibodies and the bands of interest were cropped. *n* = 3 (biological replicates). **c.** Quantification of branches number in Ctrl, Δtb-GFP and Δtb-TBPH. *n* = 15. **d.** Quantification of boutons shape in Ctrl, Δtb-GFP and Δtb-TBPH. *n* = 200. **e.** Confocal images of third instar NMJ terminals in muscle 6/7 second segment stained with anti-HRP (in green) and anti-Dlg (in red) in Ctrl (*w*^1118^), Δtb-GFP (tbph^Δ23^/tbph^Δ23^;Mhc-GAL4/UAS-GFP), Δtb-TBPH (tbph^Δ23^,UAS-TBPH/tbph^Δ23^;Mhc-GAL4/+). **f.** Quantification of Dlg intensity normalized on Ctrl. *n* > 200 boutons. **g.** Confocal images of third instar NMJ terminals in muscle 6/7 second segment stained with anti-HRP (in green) and anti-GluRIIA (in red) in Ctrl, Δtb-GFP and Δtb-TBPH. **h.** Quantification of GluRIIA intensity normalized on Ctrl. *n* > 200 boutons.

**Additional file 3: Table 1.** TG repeats distribution in drosophila DLG1 gene. TG repeats distribution in drosophila DLG1 gene. In first column TG length is reported, second and third columns report chromosomal coordinates for chromosome X, while fourth column reports exonic or intronic location. **Table 2.** TG repeats distribution in human DLG1 gene. TG repeats distribution in human DLG1 gene. In first column TG length is reported, second and third columns report chromosomal coordinates for chromosome 3, while fourth column reports exonic or intronic location.

**Additional file 4. **Individual data values**.** Individual data values for: Fig. 3: pannel a; Fig. 4: pannels a,b,c; Fig. 5: pannels a,b,c; Additional file 1 Fig. S1.

## Data Availability

All data generated or analyzed during this study are included in this published article and its additional information files.
