# Supplementary figures and images for: TDP-43 promotes the formation of neuromuscular synapses through the regulation of Disc-large expression in Drosophila skeletal muscles
Source: BMC Biol. 2020 Mar 26;18:34. doi: 10.1186/s12915-020-00767-7 (PMC7099817; doi:10.1186/s12915-020-00767-7)

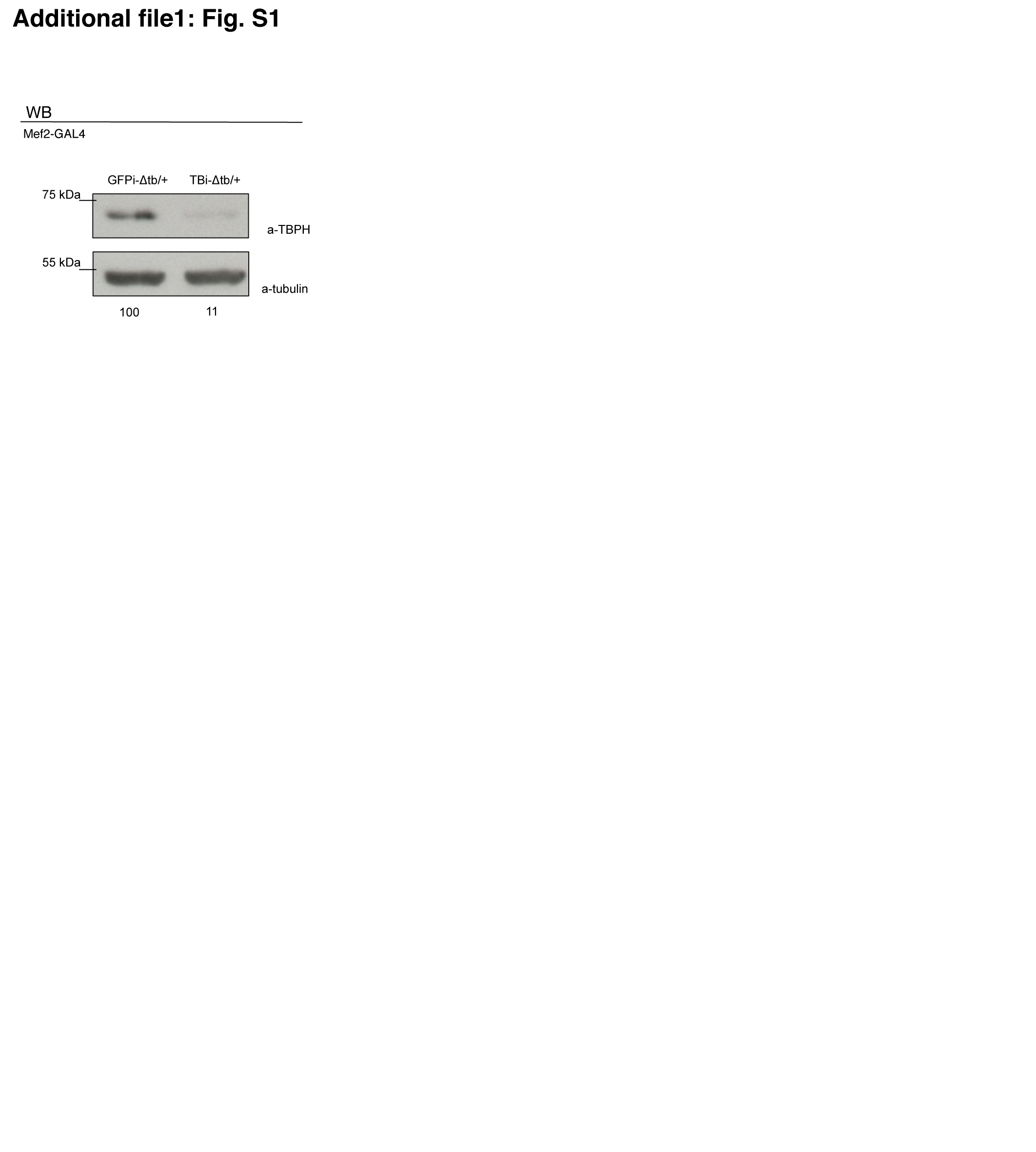

Supplement: Supplementary file 1 — Additional file 1: Figure S1. Control of TBPH silencing. Western blot analysis on larval carcasses probed for anti-TBPH and anti-tubulin in tbphΔ23/+;Mef2-GAL4/UAS-GFP-IR and tbphΔ23/+;Mef2-GAL4/UAS-TBPH-IR. The same membrane was probed with the two antibodies and the bands of interest were cropped. n = 3 (biological replicates). Individual data values are provided in the Additional file 4. Individual Data Values.xls. [file 12915_2020_767_MOESM1_ESM.tif]

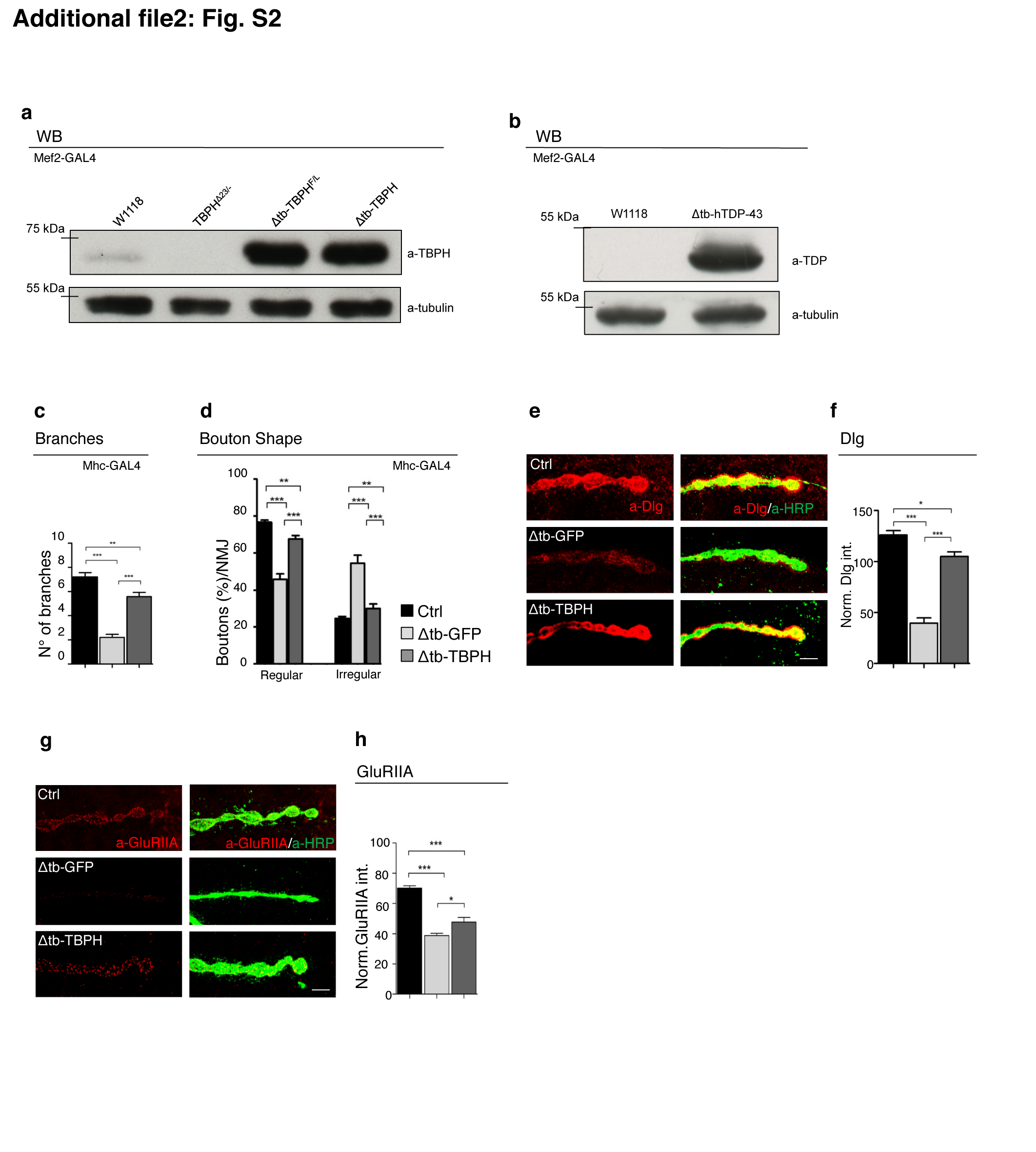

Supplement: Supplementary file 2 — Additional file 2: Figure S2. a. Western blot analysis on larval carcasses probed for anti-TBPH and anti-tubulin in Ctrl (w1118), tbphΔ23/tbphΔ23, tbphΔ23,UAS-TBPH/tbphΔ23;Mef2-GAL4/+, tbphΔ23/tbphΔ23;Mef2-GAL4/UAS-TBPHF/L. The same membrane was probed with two antibodies and the bands of interest were cropped. n = 3 (biological replicates). b. Western blot analysis on larval carcasses probed for anti-TDP and anti-tubulin in Ctrl (w1118) and tbphΔ23/tbphΔ23;Mef2-GAL4/UAS-TDP-43 The same membrane was probed with two antibodies and the bands of interest were cropped. n = 3 (biological replicates). c. Quantification of branches number in Ctrl, Δtb-GFP and Δtb-TBPH. n = 15. d. Quantification of boutons shape in Ctrl, Δtb-GFP and Δtb-TBPH. n = 200. e. Confocal images of third instar NMJ terminals in muscle 6/7 second segment stained with anti-HRP (in green) and anti-Dlg (in red) in Ctrl (w1118), Δtb-GFP (tbphΔ23/tbphΔ23;Mhc-GAL4/UAS-GFP), Δtb-TBPH (tbphΔ23,UAS-TBPH/tbphΔ23;Mhc-GAL4/+). f. Quantification of Dlg intensity normalized on Ctrl. n > 200 boutons. g. Confocal images of third instar NMJ terminals in muscle 6/7 second segment stained with anti-HRP (in green) and anti-GluRIIA (in red) in Ctrl, Δtb-GFP and Δtb-TBPH. h. Quantification of GluRIIA intensity normalized on Ctrl. n > 200 boutons. [file 12915_2020_767_MOESM2_ESM.tif]
