# Supplementary material for: TDP-43 promotes the formation of neuromuscular synapses through the regulation of Disc-large expression in Drosophila skeletal muscles
Source: BMC Biol. 2020 Mar 26;18:34. doi: 10.1186/s12915-020-00767-7 (PMC7099817; doi:10.1186/s12915-020-00767-7)
Supplement: Supplementary file 3 — Additional file 3: Table 1. TG repeats distribution in drosophila DLG1 gene. TG repeats distribution in drosophila DLG1 gene. In first column TG length is reported, second and third columns report chromosomal coordinates for chromosome X, while fourth column reports exonic or intronic location. Table 2. TG repeats distribution in human DLG1 gene. TG repeats distribution in human DLG1 gene. In first column TG length is reported, second and third columns report chromosomal coordinates for chromosome 3, while fourth column reports exonic or intronic location. [file 12915_2020_767_MOESM3_ESM.docx]

| **Supplementary Table1**  **TG repeats distribution in drosophila DLG1 gene** | | | |
| --- | --- | --- | --- |
|  |  |  |  |
|  | **X chromosome coordinates** | | |
| **(TG)10** | 11370775 | 11370794 | intronic |
| **(TG)8** | 11378800 | 11378815 | intronic |
| **(TG)7** | 11375369 | 11375382 | intronic |
| **(TG)6** | 11384976 | 11384987 | intronic |
| **(TG)5** | 11386815 | 11386824 | intronic |
|  | 11393120 | 11393129 | intronic |
| **(TG)4** | 11370135 | 11370142 | intronic |
|  | 11370872 | 11370879 | intronic |
|  | 11378740 | 11378747 | intronic |
|  | 11386805 | 11386812 | intronic |
|  | 11390049 | 11390056 | intronic |
|  | 11399008 | 11399015 | intronic |
|  | 11399710 | 11399717 | intronic |
| **(TG)3** | 11369806 | 11369811 | intronic |
|  | 11370011 | 11370016 | intronic |
|  | 11370044 | 11370049 | intronic |
|  | 11370127 | 11370132 | intronic |
|  | 11370210 | 11370215 | intronic |
|  | 11370218 | 11370223 | intronic |
|  | 11370313 | 11370318 | intronic |
|  | 11370424 | 11370429 | intronic |
|  | 11370758 | 11370763 | intronic |
|  | 11370884 | 11370889 | intronic |
|  | 11371167 | 11371172 | intronic |
|  | 11371201 | 11371206 | intronic |
|  | 11371561 | 11371566 | intronic |
|  | 11372300 | 11372305 | intronic |
|  | 11373043 | 11373048 | exonic |
|  | 11373677 | 11373682 | intronic |
|  | 11375356 | 11375361 | intronic |
|  | 11379129 | 11379134 | intronic |
|  | 11380391 | 11380396 | intronic |
|  | 11381204 | 11381209 | intronic |
|  | 11385005 | 11385010 | intronic |
|  | 11385361 | 11385366 | intronic |
|  | 11390016 | 11390034 | intronic |
|  | 11390214 | 11390215 | exonic |
|  | 11397411 | 11397416 | intronic |
|  | 11398996 | 11399001 | intronic |
|  | 11404421 | 11404426 | intronic |
|  | 11404914 | 11404919 | intronic |
|  | 11405415 | 11405420 | exonic |

**Supplementary Table 1**

TG repeats distribution in drosophila DLG1 gene. In first column TG lenght is reported, second and third columns report chromosomal coordinates for chromosome X, while fourth column reports exonic or intronic location.

| **Supplementary Table 2**  **TG repeats distribution in human DLG1 gene** | | | | | |
| --- | --- | --- | --- | --- | --- |
|  | | **3rd chromosome coordinates** | | | |
| **(TG)20** | 197051150 | | 197051252 | intronic |  |
| **(TG)19** | 197195021 | | 197195058 | intronic |  |
| **(TG)13** | 197053123 | | 197053148 | intronic |  |
| **(TG)12** | 197197412 | | 197197435 | intronic |  |
| **(TG)6** | 197147425 | | 197147436 | intronic |  |
| **(TG)5** | 197058155 | | 197058164 | intronic |  |
|  | 197058188 | | 197058197 | intronic |  |
|  | 197073579 | | 197073588 | intronic |  |
|  | 197274600 | | 197274609 | intronic |  |
| **(TG)4** | 197060359 | | 197060366 | intronic |  |
|  | 197066394 | | 197066401 | intronic |  |
|  | 197068240 | | 197068247 | intronic |  |
|  | 197086055 | | 197086062 | intronic |  |
|  | 197123665 | | 197123672 | intronic |  |
|  | 197166244 | | 197166251 | intronic |  |
|  | 197174163 | | 197174170 | intronic |  |
|  | 197179053 | | 197179060 | intronic |  |
|  | 197184329 | | 197184336 | intronic |  |
|  | 197185440 | | 197185447 | intronic |  |
|  | 197212649 | | 197212656 | intronic |  |
|  | 197276837 | | 197276844 | intronic |  |
|  | 197291687 | | 197291694 | intronic |  |
|  | 197299252 | | 197299259 | exonic |  |
| **(TG)3** | 197045242 | | 197045247 | intronic |  |
|  | 197045257 | | 197045262 | exonic |  |
|  | 197046278 | | 197046283 | intronic |  |
|  | 197047625 | | 197047630 | intronic |  |
|  | 197048316 | | 197048321 | intronic |  |
|  | 197049503 | | 197049508 | intronic |  |
|  | 197051556 | | 197051561 | intronic |  |
|  | 197052665 | | 197052670 | intronic |  |
|  | 197055326 | | 197055331 | intronic |  |
|  | 197056235 | | 197056240 | intronic |  |
|  | 197056471 | | 197056476 | intronic |  |
|  | 197056566 | | 197056571 | intronic |  |
|  | 197057421 | | 197057426 | intronic |  |
|  | 197057558 | | 197057563 | intronic |  |
|  | 197058202 | | 197058207 | intronic |  |
|  | 197060096 | | 197060101 | intronic |  |
|  | 197064146 | | 197064151 | intronic |  |
|  | 197068155 | | 197068160 | intronic |  |
|  | 197068197 | | 197068202 | intronic |  |
|  | 197069758 | | 197069763 | intronic |  |
|  | 197070528 | | 197070533 | intronic |  |
|  | 197070632 | | 197070637 | intronic |  |
|  | 197072075 | | 197072080 | intronic |  |
|  | 197073613 | | 197073618 | intronic |  |
|  | 197079698 | | 197079703 | intronic |  |
|  | 197081432 | | 197081437 | intronic |  |
|  | 197082346 | | 197082351 | intronic |  |
|  | 197082852 | | 197082857 | intronic |  |
|  | 197085094 | | 197085099 | intronic |  |
|  | 197085748 | | 197085753 | intronic |  |
|  | 197086555 | | 197086560 | intronic |  |
|  | 197087305 | | 197087310 | intronic |  |
|  | 197087917 | | 197087922 | intronic |  |
|  | 197088309 | | 197088314 | intronic |  |
|  | 197089053 | | 197089058 | intronic |  |
|  | 197091040 | | 197091045 | intronic |  |
|  | 197093821 | | 197093826 | intronic |  |
|  | 197096916 | | 197096921 | intronic |  |
|  | 197099325 | | 197099330 | intronic |  |
|  | 197101144 | | 197101149 | intronic |  |
|  | 197101629 | | 197101634 | intronic |  |
|  | 197105420 | | 197105425 | intronic |  |
|  | 197108387 | | 197108392 | intronic |  |
|  | 197115417 | | 197115422 | intronic |  |
|  | 197115523 | | 197115528 | intronic |  |
|  | 197116759 | | 197116764 | intronic |  |
|  | 197119165 | | 197119170 | intronic |  |
|  | 197124954 | | 197124959 | intronic |  |
|  | 197127690 | | 197127695 | intronic |  |
|  | 197129391 | | 197129396 | intronic |  |
|  | 197130235 | | 197130240 | intronic |  |
|  | 197130940 | | 197130945 | intronic |  |
|  | 197131918 | | 197131923 | intronic |  |
|  | 197132560 | | 197132565 | intronic |  |
|  | 197145010 | | 197145015 | intronic |  |
|  | 197146794 | | 197146799 | intronic |  |
|  | 197146804 | | 197146809 | intronic |  |
|  | 197149343 | | 197149348 | intronic |  |
|  | 197153027 | | 197153032 | intronic |  |
|  | 197155351 | | 197155356 | intronic |  |
|  | 197156315 | | 197156320 | intronic |  |
|  | 197162333 | | 197162338 | intronic |  |
|  | 197162628 | | 197162633 | intronic |  |
|  | 197163444 | | 197163449 | intronic |  |
|  | 197163649 | | 197163654 | intronic |  |
|  | 197165788 | | 197165793 | intronic |  |
|  | 197166776 | | 197166781 | intronic |  |
|  | 197169968 | | 197169973 | intronic |  |
|  | 197170454 | | 197170459 | intronic |  |
|  | 197170464 | | 197170469 | intronic |  |
|  | 197170898 | | 197170903 | intronic |  |
|  | 197178440 | | 197178445 | intronic |  |
|  | 197178977 | | 197178982 | intronic |  |
|  | 197185513 | | 197185518 | intronic |  |
|  | 197186203 | | 197186208 | intronic |  |
|  | 197190365 | | 197190370 | intronic |  |
|  | 197191841 | | 197191846 | intronic |  |
|  | 197192088 | | 197192093 | intronic |  |
|  | 197195987 | | 197196037 | intronic |  |
|  | 197199437 | | 197199442 | intronic |  |
|  | 197200877 | | 197200882 | intronic |  |
|  | 197207337 | | 197207342 | intronic |  |
|  | 197207770 | | 197207775 | intronic |  |
|  | 197208052 | | 197208057 | intronic |  |
|  | 197216894 | | 197216899 | intronic |  |
|  | 197225265 | | 197225270 | intronic |  |
|  | 197227165 | | 197227170 | intronic |  |
|  | 197231172 | | 197231177 | intronic |  |
|  | 197234181 | | 197234186 | intronic |  |
|  | 197238426 | | 197238431 | intronic |  |
|  | 197246353 | | 197246358 | intronic |  |
|  | 197246977 | | 197246982 | intronic |  |
|  | 197249355 | | 197249360 | intronic |  |
|  | 197254002 | | 197254007 | intronic |  |
|  | 197255401 | | 197255406 | intronic |  |
|  | 197265147 | | 197265152 | intronic |  |
|  | 197279016 | | 197279021 | intronic |  |
|  | 197280957 | | 197280962 | intronic |  |
|  | 197294012 | | 197294017 | intronic |  |
|  | 197299254 | | 197299259 | intronic |  |

**Supplementary Table 2**

TG repeats distribution in human DLG1 gene. In first column TG lenght is reported, second and third columns report chromosomal coordinates for chromosome 3, while fourth column reports exonic or intronic location.
